# A diffuse large B cell lymphoma emerging with breast cancer relapse

**DOI:** 10.11604/pamj.2018.31.125.15057

**Published:** 2018-10-19

**Authors:** Abdulkadir Karismaz, Mehmet Hilmi Dogu, Gülben Huq, Sermin Altindal, Osman Yokus, Elif Suyani

**Affiliations:** 1 University of Health Sciences, Istanbul Training and Research Hospital, Department of Hematology, Istanbul, Turkey; 2 University of Health Sciences, Istanbul Training and Research Hospital, Department of Pathology, Istanbul, Turkey

**Keywords:** Breast cancer, lymphoma, therapy-related hematological malignancies

## Abstract

The prevalence of secondary cancers associated with the breast cancer treatment has increased, which is due to the administration of cytotoxic/hormonal drugs as well as radiotherapy. A 54-year-old female patient with a history of breast cancer for 4 years and receiving tamoxifen the hematology clinic with fatigue and nosebleed. Laboratory parameters were revealed pancytopenia. The bone marrow biopsy finding was compatible with CD20 positive high-grade B cell lymphoma resembling diffuse large B cell lymphoma. The patient started to receive a chemotherapy. Her hemogram values displayed an improvement after the second cycle. However, interim PET-BT, performed after the fourth cycle, showed an incomplete response in cervical lymphatic nodes. Then, a tru-cut biopsy was performed resulting in breast cancer metastasis. This is an unusual case of secondary-DLBCL presenting with pancytopenia and occuring 4 years after the diagnosis of breast cancer. In conclusion, clinicians should carefully set the dosage of chemotherapy drugs to avoid the long-term side effects associated with such drugs.

## Introduction

Breast cancer is among the most common tumors occurring in women, and recently, survival rates of the patients have progressively improved owing to neoadjuvant, adjuvant, endocrine and targeted therapies. However, chemotherapy-associated long-term side effects have gradually been on the rise in these patients. Adjuvant chemotherapies comprising particularly alkylating agents and topoisomerase inhibitors may increase the risk of leukemia especially in patients with early-stage breast cancer [1-3]. Prophylactic or adjuvant chemotherapy administered due to early-stage breast cancer is demonstrated to increase 10-year hematological cancer risk by 0.5%, which is twice higher than those reported in previous studies [4]. While the most prevalent hematological cancers associated with the treatment are acute myeloid leukemia (AML) and myelodysplastic syndrome (MDS) [2, 3], treatment-associated lymphomas constitute quite a rare clinical picture [5, 6]. This report presents a secondary diffuse large B-cell lymphoma emerging with breast cancer relapse.

## Patient and observation

**Figure 1:**
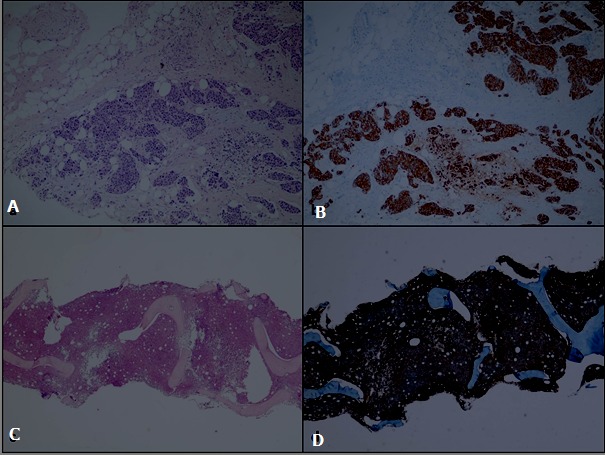
A) carcinoma infiltration in solid islands wiping the lymph node structure; B) diffuse and strong pankeratin positivity in carcinoma cells; C) extensive infiltration in the bone marrow, presenting a diffuse pattern, consisting of cells in large centroblastic morphology; D) extensive and strong CD20 expression in the bone marrow infiltration

A 54-year-old female patient referred to the hematology clinic with fatigue and nosebleed. She had a history of type-2 diabetes mellitus and breast cancer. The patient was diagnosed with breast cancer four years ago and underwent a mastectomy followed by radiotherapy and chemotherapy, and she has been receiving tamoxifen since that time. The chemotherapy protocol was inaccessible because the chemotherapy was administered in Syria. The physical examination revealed a swollen right arm, ecchymoses in arms, and fixed, firm, painless cervical lymphadenopathies, the largest of which was 2x2cm in size and located on the right. Laboratory parameters were as follows: white blood cell count: 3.62 x 10^9^/L with 0.84x10^9^/L neutrophils, hemoglobin: 11 g/dL, platelet count: 10x10^9^/L. Biochemical, liver and renal function test results were normal. HBsAg, Anti-HBS, Anti-HCV and Anti-HIV test results were negative. Platelet count by peripheral smear was compatible with the hemogram count without any atypical cells. The lymph node biopsy could not be performed since the platelet count did not rise despite transfusion. Instead, bone marrow aspiration and biopsy were performed for diagnostic purposes. The biopsy finding was compatible with CD20 positive high-grade B cell lymphoma resembling diffuse large B cell lymphoma (Figure 1 (A, B)). Body ^18^Fluorodeoxyglucose positron emission tomography/computed tomography (^18^F-FDG PET/CT) performed for staging revealed following lymphadenopathies: 1.3x0.8cm-sized at the right parotid gland inferior (SUVmax: 11.8), right inferior jugular area (SUVmax: 13.1), and 2x1.5 cm-sized (largest) (SUVmax: 6.8) in the right deep cervical location. The patient started to receive a chemotherapy protocol combining rituximab-cyclophosphamide-doxorubicin-vincristine-prednisolone (R-CHOP). Her hemogram values displayed an improvement after the second cycle. However, interim PET-BT, performed after the fourth cycle, showed an incomplete response in cervical lymphatic nodes. Then, a tru-cut biopsy was performed from the lymph node in the neck as blood values were better. The patient continued to receive R-CHOP until the biopsy result was obtained. R-CHOP was stopped at the sixth cycle. The lymph node biopsy result showed breast cancer metastasis (Figure 1 (C, D)). She underwent a bone marrow biopsy after six cycles of R-CHOP, which revealed that the lymphoma infiltration disappeared. The patient's breast cancer treatment continues.

## Discussion

Breast cancer is one of the most common causes of cancer-related deaths in women. Although the mortality rate related to breast cancer has decreased lately with the development of early diagnosis and treatment techniques, the prevalence of secondary cancers associated with the treatment has increased due to the administration of cytotoxic/hormonal drugs as well as radiotherapy [2, 3]. Regarding the hematological malignancies, lymphomas are encountered rarely compared to AML and MDS in those patients [2, 3]. Given the high prevalence of breast cancer, these findings indicate that the number of patients under the risk of secondary cancer triggered by treatment might be actually higher than expected. The secondary cancers associated with breast cancer chemotherapy are largely associated with alkylating agents and topoisomerase II inhibitors [7]. Alkylating agents cause gene mutations and deletions in the long arms of chromosomes 5 and 7 by cross linking with DNA. Those gene mutations affect IL-3, IL-4 and other genes which are active in cell proliferation. Deletions, in turn, may activate RAS and trigger tumor suppressing gene mutations, which cause excessive cell proliferation, differentiation, and eventually leukemia [8]. Topoisomerase II inhibitors create a complex with DNA and topoisomerase II enzyme, block the enzyme activity and result in broken DNA fibers. Those broken fibers cause a rearrangement mostly in the acute lymphoblastic leukemia (ALL)-1 gene site and DNA damage, which may thus end in leukemia [9]. Similar events constitute the underlying mechanism of secondary lymphomas in patients who are applied breast cancer chemotherapy. And secondary lymphomas are usually expected to occur 5 years after the initial therapy [10]. This is an unusual case of secondary-DLBCL presenting with pancytopenia and occurring 4 years after the diagnosis of breast cancer. Since presence of pancytopenia in a breast cancer patient usually suggests the development of either leukemia or bone marrow metastases, a bone marrow biopsy showing lymphoma infiltration was astonishing. Also, the lymph nodes were involved with breast cancer unexpectedly. It was critically important to perform the sampling to distinguish it from lymphoma and make the treatment decision on this difference. And other important issue is the timing of the lymphoma diagnosis that is 4 years after the breast cancer diagnosis and probably less than 4 years after the treatment, leading to raise the doubt whether the patient's lymphoma was secondary or already existed at the time of diagnosis of the breast cancer.

## Conclusion

Clinicians should carefully set the dosage of chemotherapy drugs, especially topoisomerase II inhibitors and alkylating agents, to avoid the long-term side effects associated with such drugs.

## Competing interests

The authors declare no competing interests.

## References

[1] Wolff AC, Blackford AL, Visvanathan K (2015). Risk of marrow neoplasms after adjuvant breast cancer therapy: the national comprehensive cancer network experience. J Clin Oncol.

[2] Bernard-Marty C, Mano M, Paesmans M (2003). Second malignancies following adjuvant chemotherapy: 6-year results from a Belgian randomized study comparing cyclophosphamide, methotrexate and 5-fluorouracil (CMF) with an anthracycline-based regimen in adjuvant treatment of node-positive breast cancer patients. Ann Oncol.

[3] Praga C, Bergh J, Bliss J, Bonneterre J, Cesana B, Coombes RC (2005). Risk of acute myeloid leukemia and myelodysplastic syndrome in trials of adjuvant epirubicin for early breast cancer: correlation with doses of epirubicin and cyclophosphamide. J Clin Oncol.

[4] Fisher B, Rockette H, Fisher ER (1985). Leukemia in breast cancer patients following adjuvant chemotherapy or postoperative radiation: the NSABP experience. J Clin Oncol.

[5] Rossi D, Sarti D, Malerba L (2016). Secondary bone marrow malignancies after adjuvant chemotherapy for breast cancer: a report of 2 cases and a review of the literature. Tumori.

[6] Zhang B, Zhang X, Li M, Kong L, Deng X, Yu J (2016). How breast cancer chemotherapy increases the risk of leukemia: thoughts about a case of diffuse large B-cell lymphoma and leukemia after breast cancer chemotherapy. Cancer Biol Ther.

[7] Yagita M, Ieki Y, Onishi R (1998). Therapy-related leukemia and myelodysplasia following oral administration of etoposide for recurrent breast cancer. Int J Oncol.

[8] Takemoto Y, Hata T, Kamino K (1995). Leukemia developing after 131I treatment for thyroid cancer in a patient with Werner's syndrome: molecular and cytogenetic studies: molecular and cytogenetic studies. Intern Med.

[9] Felix CA, Hosler MR, Winick NJ, Masterson M, Wilson AE, Lange BJ (1995). ALL-1 gene rearrangements in DNA topoisomerase II inhibitor-related leukemia in children. Blood.

[10] Krishnan B, Morgan GJ (2007). Non-Hodgkin lymphoma secondary to cancer chemotherapy. Cancer Epidemiol Biomarkers Prev.

